# Circular RNAs in peripheral blood mononuclear cells are more stable than linear RNAs upon sample processing delay

**DOI:** 10.1111/jcmm.17525

**Published:** 2022-08-30

**Authors:** Guoxia Wen, Wanjun Gu

**Affiliations:** ^1^ State Key Laboratory of Bioelectronics, School of Biological Sciences and Medical Engineering Southeast University Nanjing China; ^2^ Collaborative Innovation Center of Jiangsu Province of Cancer Prevention and Treatment of Chinese Medicine Nanjing University of Chinese Medicine Nanjing China; ^3^ School of Artificial Intelligence and Information Technology Nanjing University of Chinese Medicine Nanjing China

**Keywords:** differential alternative splicing, differential gene expression, PBMC circRNAs, sample processing delay

## Abstract

Circular RNAs (circRNAs) are a novel class of RNAs with closed loop structure. Blood circRNAs are widely acknowledged to be more stable than linear mRNAs, which show promising prospect to be liquid biopsy biomarkers for clinical applications. However, accumulating studies have demonstrated that sample processing delays have profound effects on blood transcriptome expression profiles, wherein knowledge remains elusive about the impacts of prolonged sample processing on blood expression profiles of circRNAs. We collected whole blood samples from three donors and isolated peripheral blood mononuclear cells (PBMCs) at six different incubation time points. We measured total RNA expression profiles using RNA sequencing (RNA‐seq) and investigated the differentially expressed circRNAs, mRNAs and lncRNAs upon blood processing delay. Meanwhile, we explored the underlying inducement of aberrant expression of circRNAs against their corresponding mRNA transcripts. Finally, we utilized *rMATS‐turbo* and *CIRI‐AS*, respectively, to screen out differential alternative splicing (AS) events in linear mRNAs and circRNAs. Sample incubation at 4°C lasting to 48 hours (h) led to minimal effects to circRNAs' expression. However, it induced extensive alterations for mRNAs and lncRNAs when the incubation time was beyond 12 h. Additionally, only 2 h processing delays may result in profound impacts on AS events of linear mRNAs, while less impact on the equivalence of circRNAs. Our results suggested that PBMC circRNAs are stable upon sample processing delay, which are more suitable to be liquid biopsy biomarkers.

## 1 INTRODUCTION

In liquid biopsy, many blood RNA biomarkers have been increasingly proposed to the diagnosis, prognosis and treatment guidance of human diseases.^1^, ^2^, ^3^ However, the clinical translation of these RNA biomarkers has been hindered by the complex procedures of sample processing.^4^ Recent studies have suggested that several steps in blood RNA processing are likely to introduce technical variations, ranging from blood collection,^5^, ^6^ leukocyte isolation method^5^, ^7^ and preserved temperature^5^, ^7^ to incubation time.^8^, ^9^, ^10^, ^11^ Therefore, some standard operating procedures have been introduced in blood sample processing,^12^ including the use of commercial blood collection tubes, extraction kits, portable devices and automated workstations.^13^, ^14^, ^15^ The standardization of these steps has greatly improved the whole procedure of blood sample processing, which may reduce technical noises and increase data reproducibility.^12^ However, the incubation time from blood draw to RNA extraction is hardly to be standardized due to the limitation of working time, location and other situations.^10^ The prolonged storage of blood samples has been observed to make substantial changes on the measured blood transcriptomes.^8^, ^9^, ^10^, ^11^ For example, Dvinge et al. performed bulk RNA‐seq of blood samples from four healthy donors, and found rapid transcriptional and post‐transcriptional changes upon different blood incubation times at room temperature or cryopreservation.^8^ In addition, Massoni‐Badosa et al. performed single‐cell RNA‐seq and single‐cell ATAC‐seq of human blood samples from two healthy donors and three leukaemia patients. They also concluded that ex vivo prolonged blood storage induced marked alterations of transcriptional profiles and chromatin accessibility at the single‐cell level.^9^ Similarly, Savage et al. performed multi‐omic profiling of human peripheral blood samples at different handling time points and investigated the effect of delayed blood processing on the multi‐omic datasets, including targeted bulk PBMCs transcriptomics, PBMC single‐cell transcriptomics, cell numbers and plasma proteomes.^10^ They found extensive changes of single‐cell transcriptome and plasma proteome after 4 h incubation, while accumulating differences were observed for the targeted bulk transcriptomes and the number of immune cells during an overnight rest (18 h) after blood draw.^10^ All these studies indicated that blood mRNA transcripts were sensitive to handing delays, which may confound the biological findings from blood mRNA expression experiments and the translation of blood mRNA signatures for disease management.^8^, ^9^

To overcome this limitation, many recent studies have proposed blood circRNAs as a new kind of blood RNA biomarkers for human diseases.^16^ Unlike linear mRNAs, circRNAs have a unique circular structure that lacks free ends, poly(A) tails and 5′ caps.^17^ This makes circRNAs resistant to de‐adenylation, decapping and exonucleases.^17^ Therefore, circRNAs are observed to be more stable than linear mRNA transcripts.^18^, ^19^ Specifically, the median half‐life of circRNAs was at least 2.5 times longer than that of their linear mRNA counterparts in mammary cells.^18^ Additionally, the expression levels of circRNAs in serum exosomes were stable for serum samples that incubated at room temperature for up to 24 h.^19^ These results hinted that blood circRNA expressions should be more robust than linear mRNAs under different blood incubation times. In contrast, Rochow et al. found gradually reduced expression values of circRNAs in kidney cancer tissue and cell lines with RIN (RNA integrity number) value reduction.^20^ They suggested that circRNAs were subjected to degradative processes in clinical samples, which was similar to linear mRNAs.^20^ The different conclusions made in previous studies make it difficult to predict how circRNAs will respond to blood incubation times in sample processing. Therefore, there is still an urgent need to investigate the effect of blood sample processing delays on circRNA expression profiles.

In this study, we measured the expression profiles of linear mRNAs, long non‐coding RNAs (lncRNAs) and circRNAs, in human PBMC samples with varying blood incubation times in blood sample processing. We tried to answer the following two questions. First, what are the expression changes of human PBMC mRNAs, lncRNAs and circRNAs in blood samples at different incubation time points? Second, are there any differences between PBMC circRNA and linear RNA changes upon blood incubation time? This will help us gain some insights of circRNAs expression in human blood samples, which is especially important for advancing PBMC RNA biomarkers in liquid biopsy of human diseases.

## 2 MATERIALS AND METHODS

### 2.1 Blood collection, PBMC isolation and RNA extraction

We recruited three healthy donors from the First Affiliated Hospital of Nanjing Medical University. All individuals participated anonymously in consideration of privacy and security concerns. First, 30 millilitres peripheral blood samples were collected into six *PAXgene Blood RNA Tubes* (*BD*, NJ, USA) from each volunteer by venipuncture. These blood samples were immediately preserved at 4°C for 0 h, 2 h, 6 h, 12 h, 24 h and 48 h, respectively, before PBMCs isolation. Next, PBMCs were isolated by applying *Ficoll*‐*Paque Premium* (*GE Healthcare*, IL, USA) according to the manufacturer's instructions. Then, total RNAs were extracted from PBMCs using the *TRIzol* reagent (*Invitrogen*, CA, USA) and purified with the *mirVana RNA Isolation Kit* (*Ambion*, MA, USA). Finally, 1% formaldehyde denaturing gel electrophoresis was used to monitor RNA degradation and contamination. The RNA integrity was measured by *Agilent 2100 Bioanalyzer* (*Agilent Technologies*, CA, USA).

This study was performed in accordance with the principles outlined in the Declaration of Helsinki.

### 2.2 RNA‐seq library preparation and sequencing

A total amount of 3 μg RNA per sample were used as the starting material for library construction. First, ribosomal RNAs were removed from total RNAs by *Epicentre Ribo‐zeroTM rRNA Removal Kit* (*Epicentre*, USA). Then, the remaining RNA samples were used for library construction by *NEBNext Ultra™ Directional RNA Library Prep Kit* (*New England Lab*, MA, USA). In total, we constructed 18 rRNA‐depleted strand‐specific RNA‐seq libraries (6 RNA‐seq libraries for each healthy donors). Finally, all these ribo‐depleted RNA‐seq libraries were sequenced on *Illumina HiSeq X Ten* platform (*Illumina*, CA, USA) using paired‐end 150 bp runs.

### 2.3 Expression quantification of PBMC RNA transcripts

For each RNA‐seq dataset, we identified the expressed circRNA transcripts using *CIRI‐full* ^21^ with *GRCh38* reference genome, *Ensembl* 94 gene annotation and the default parameters. Next, we constructed a reference library of expressed PBMC circular transcripts by combining the de novo constructed circular transcripts in annotated human genes from *CIRI‐full* output and the known blood circRNA transcripts from *isoCirc* catalogue.^22^ Then, we quantified the expression values of both circular and linear RNA transcripts using *AQUARIUM* ^23^ with the compiled reference library of circular transcripts, *Ensembl* 94 gene annotation and the default parameters. We chose to use *AQUARIUM* for RNA expression quantification, since we have observed its superior performance in estimating the expression values of both circular and linear RNAs at the transcript level.^23^ After calculating the transcripts per million (TPM) values for all linear and circular RNA transcripts in each RNA‐seq dataset, we integrated all the expressed transcripts in 18 RNA‐seq datasets. Finally, those lowly expressed transcripts (a transcript that has a TPM value smaller than one in more than four samples) were excluded for further analysis. For circRNAs, the transcripts whose biotypes of parental genes were not protein coding or lncRNAs based on *Ensembl* 94 gene annotation were further filtered out.

### 2.4 Differential expression analysis

To investigate the transcriptome changes between different blood incubation time points, we imported the transcript expression profiles from *AQUARIUM* output using *tximport* ^24^ and calculated the expression differences of both circular and linear RNA transcripts using *DESeq2* with likelihood ratio test and *apeglm* shrinkage method.^25^ We chose *DESeq2* for differential expression analysis, since it has better performance for alignment‐free isoform quantification tools.^26^ Transcripts or genes with |log_2_(fold change)| > 0.5 and adjusted *p*‐value < .05 were considered as significantly differential expression. Among them, some were further classified into newborn or degraded transcripts, which suggests the dynamic gain or loss of RNA transcripts upon incubation. We defined the transcripts with a TPM value larger than one in at least two replicated samples at a time point as the expressed transcripts of that time point. Transcripts that were expressed at the examined time points but not expressed at 0 h were defined as the newborn transcripts. Similarly, transcripts that were expressed at 0 h but not expressed at the examined time points were defined as the degraded transcripts.

To explore whether circRNA expression changes induced in the course of sample handling delay were caused by the alterations of cell populations in the blood samples, we ran Cell Fraction analysis module in CIBERSORTx^27^ to deconvolve immune cell subsets from PBMC samples using our bulk RNA‐seq data. The proportions of 12 immune cell subsets in each PBMC sample at all six time points were estimated by using LM22 signature as the reference matrix. The statistical significance of the alterations of the proportion of each immune cell over incubation time was computed by *Kruskal–Wallis* test. To clarify whether the dynamic changes of circRNAs, mRNAs and lncRNAs were relevant to the viability of samples, we screened the mitochondrial RNAs (mtRNAs) from our RNA‐seq data based on *Ensembl* 94 annotation, and then, we calculated the total expression of mtRNAs of three samples at each time point and performed multiple comparison of mtRNAs content by using *anova* test.

### 2.5 Differential alternative splicing analysis

Other than expression abundance, alternative splicing (AS) event can produce various RNA transcripts from one gene. To identify the changes of AS events between PBMC samples at different incubation time points, we first detected the AS events of linear mRNAs and circRNAs using *rMATS‐turbo* ^28^ and *CIRI‐AS*,^29^ respectively. Both methods used a ratio value (*Ψ*) to estimate the inclusion possibility of a targeted exon. Next, we calculated the difference of *Ψ* values (△*Ψ*) between replicates at different incubation time points for each exon. Then, we used paired *t*‐test to calculate the *p‐*value of differential splicing events with △*Ψ*, of which a stringent threshold, *p*‐value < .05 and |△*Ψ*| ≥ .05, was adopted to define significantly differential AS. Finally, the number of abnormal AS at each time course was normalized by dividing the total number of identified AS exons of *CIRI‐AS* or *rMATS‐turbo*.

### 2.6 Functional enrichment analysis

To explore the biological functions of blood incubation‐related transcripts, we performed the Gene Set Enrichment Analysis (*GSEA*)^30^ using *gseGO()* function and visualized the enriched gene sets using *enrichplot()* function in *clusterProfiler* package.^31^ Biological pathways (BP) that have *p*‐value less than .05 were considered as significantly enriched.

## 3 RESULTS

### 3.1 Expression landscape of circular and linear RNA transcripts in human PBMC samples

**FIGURE 1:**
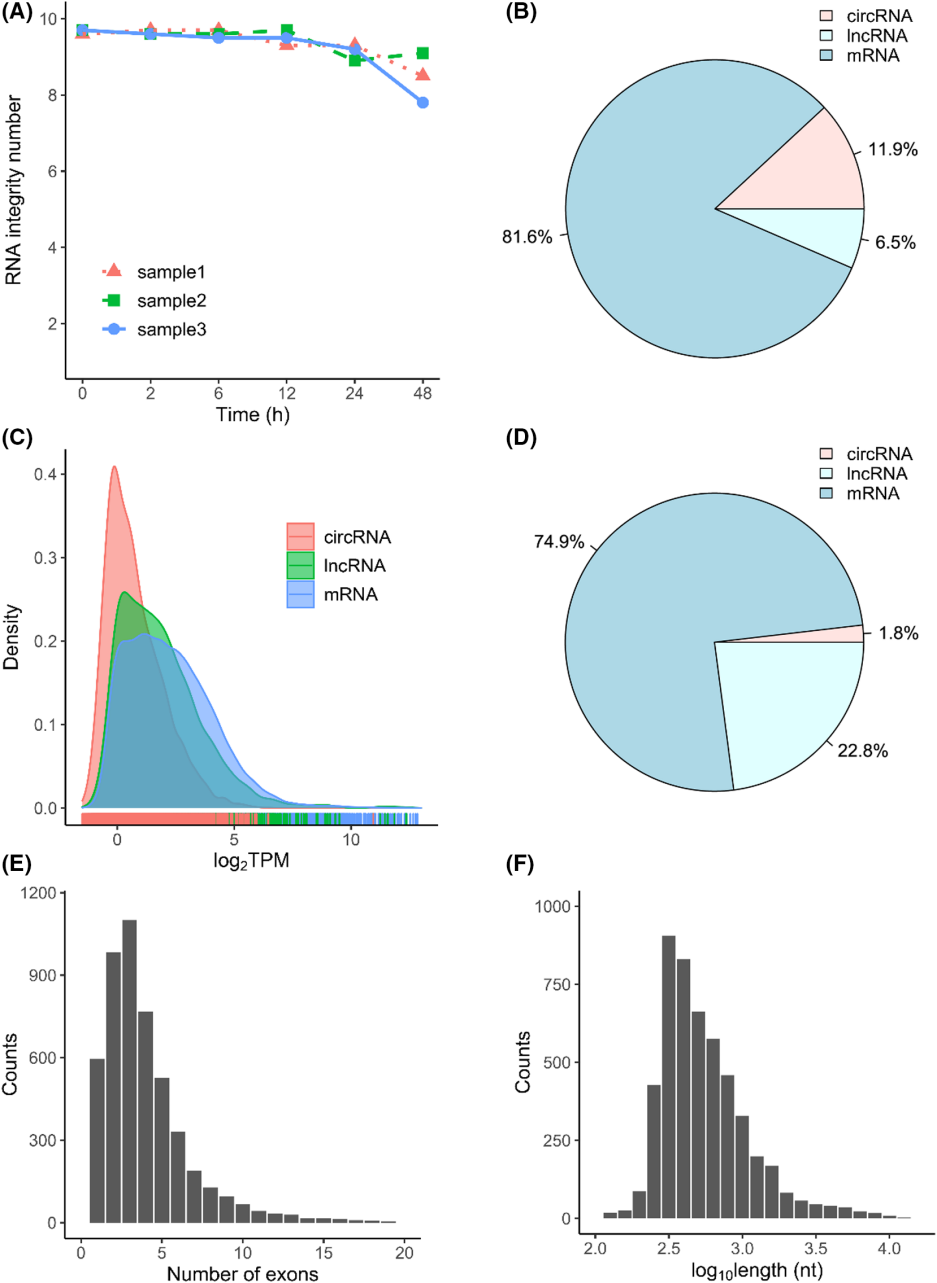
Expression landscape of circular and linear RNA transcripts in human blood samples: (A) RIN values of RNA isolated from PBMCs of all samples at six time points; (B) the fraction of circRNA, mRNA and lncRNA species; (C) the distribution of expression abundance of circRNAs, mRNAs and lncRNAs; (D) the fraction of expression values of circRNAs, mRNAs and lncRNAs; (E) the distribution of exonic number of circRNAs; and (F) the distribution of exonic circRNA length within the repertoire

Whole blood samples were collected from three healthy donors in anticoagulant blood collection tubes and were immediately incubated at 4°C for six scheduled time intervals, including 0 h, 2 h, 6 h, 12 h, 24 h and 48 h. PBMCs were subsequently isolated from these 18 samples, and total RNAs were extracted for transcriptome profiling. Although the RIN value decreases with the incubation time interval (Figure 1A), the quality of extracted RNAs was consistently good in all samples (Figure S1). The average RIN value of all these samples was at 9.3, and the sample with the lowest RNA quality had a RIN value at 7.8 (Figure 1A). These RNA samples were used for RNA‐seq library construction and transcriptome profiling. For each RNA‐seq data, circRNA transcripts were identified using a home‐built computational pipeline (see Materials and Methods). Then, the expression of circRNAs, linear mRNAs and lncRNAs were quantified. RNA transcripts with low expression abundance in these blood samples were further filtered. Additionally, 62 circular transcripts were excluded due to biotypes of corresponding genes not being protein coding or lncRNA, of which 58 were pseudogenes, 1 misc_RNA, 1 snoRNA and 2 TR genes (see Materials and Methods). Finally, a repertoire of expressed RNA transcripts in all these PBMC samples was constructed. In this PBMC transcriptome repertoire, 41,936 RNA transcripts were expressed in total. Among them, 5007 (11.9%) were circular transcripts, and 2709 (6.5%) were linear lncRNAs. The remaining 34,222 (81.6%) transcripts are coding mRNA transcripts (Figure 1B). As expected, the coding mRNA has the highest number of expressed transcripts (Figure 1B) and expression values (Figure 1D). Although lncRNAs have the smallest number of expressed transcripts, their expressed abundance (22.8%) is higher than that of circRNAs, which account only 1.8% of the total expressed RNA transcripts (Figure 1D). This can be explained by the lowest mean expression value of circRNA transcripts (Figure 1C), since most circRNAs were lowly expressed in mammalian samples.^32^ For exonic circRNAs (ecircRNAs), most were composed of no more than five exons (Figure 1E). Additionally, the length of most ecircRNAs is less than 1000 base pairs (Figure 1F). In general, this de novo constructed PBMC transcriptome has similar characteristics that were observed in previous studies.^33^, ^34^

### 3.2 Dynamic expression changes of circular and linear RNA transcripts upon incubation

**FIGURE 2:**
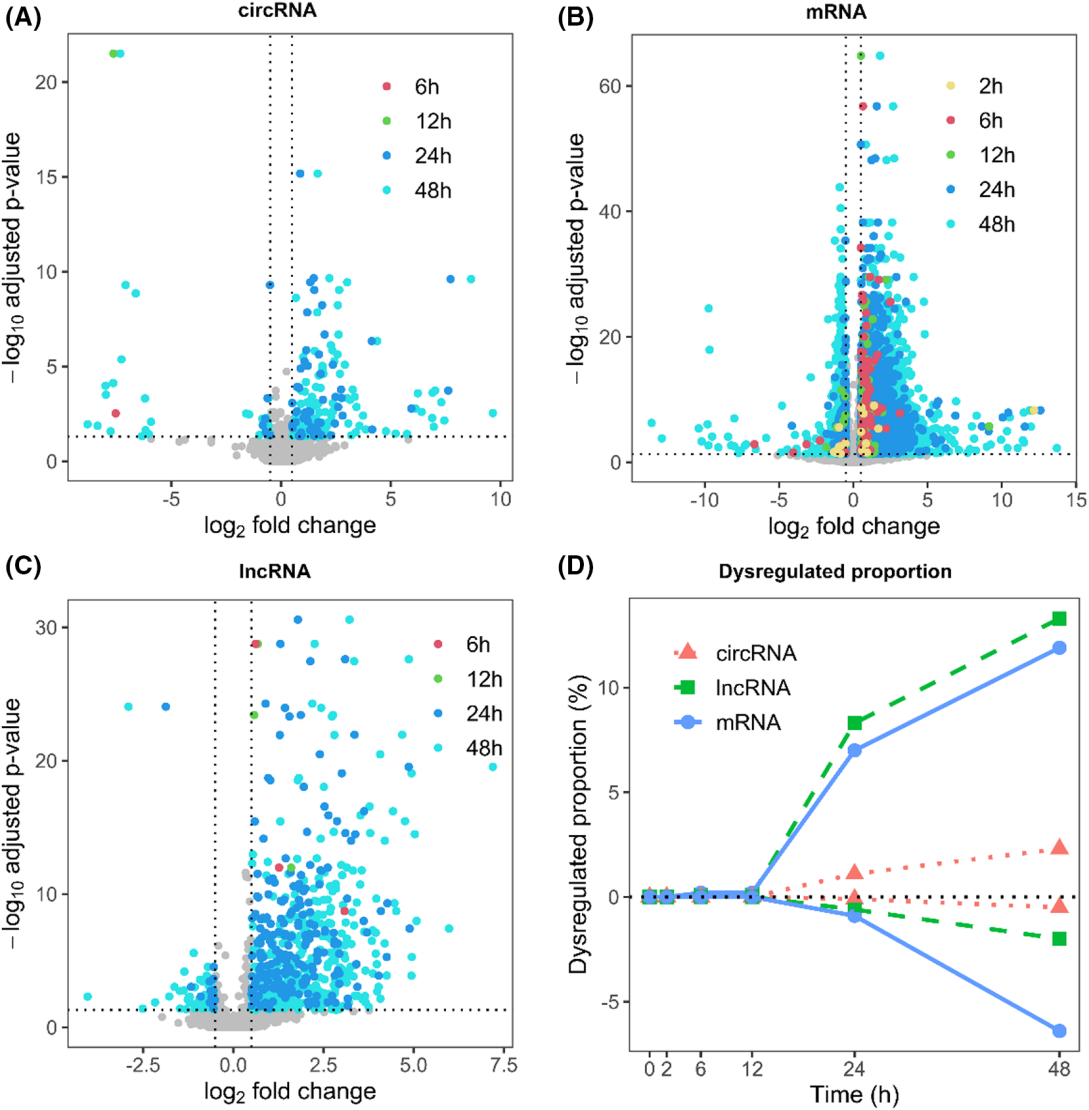
Sample processing delay induced substantial transcriptome changes. The number of dysregulated circRNAs (A), mRNAs (B) and lncRNAs (C) at 2 h (yellow), 6 h (red), 12 h (green), 24 h (blue) and 48 h (turquoise) against the first time point (0 h) was shown. Adjusted *p*‐value cut‐offs of .05 and |log_2_(fold_change)| of 0.5 were indicated by dashed lines. The proportion of dysregulated transcripts at each time course (D) was also shown

To quantify the effects of sample processing delays from blood collection to PBMC isolation on blood transcriptome, we first investigated the changes of expression levels of RNA transcripts, including mRNAs, lncRNAs and circRNAs, between the original transcriptome and those with processing delays. For each delayed processing transcriptome, we used *DESeq2* to identify the differentially expressed transcripts at each time point (2 h, 6 h, 12 h, 24 h, 48 h) against the first time point (0 h). In comparison with the transcriptome of immediate isolation (0 h), substantial transcriptome changes were observed in samples with handling delays (Figure 2A–C; Table S1). The number of dysregulated transcripts gradually increased with the time interval of sample processing (Figure 2A–C). This is consistent to the findings observed in previous studies of mRNA transcripts.^8^, ^9^, ^10^ Comparing different types of RNA transcripts, we found that circRNAs had the least number of transcripts that were dysregulated in prolonged handling procedures, while mRNAs had the largest number of induced changes (Figure 2A–C). This is still the case when the changes were normalized to the proportion of dysregulated transcripts by dividing the total number of transcripts in the class (Figure 2D). Taken together, our results suggested that blood handling delays within 12 h had relatively small effects on the expression abundance of linear RNA transcripts. However, linear RNA transcripts, both mRNAs and lncRNAs, experienced massive expression changes when the samples were handled beyond 12 h. In comparison, prolonged blood handling had the smallest effects on circRNA expressions, even for the samples with the time interval as long as 48 h. Additionally, we explored whether these expression changes were caused by the alterations of immune cell population over time. We observed no significant differences of cell proportion at six time points for almost all cell types, excluding the resting CD4 memory T cell (Figure S2). This suggests that the induced transcriptomic changes upon blood handling delay were not likely to be caused by cell population alteration. Meanwhile, we observed there was no statistical difference of total content of mtRNAs of three samples at each time point (Figure S3), indicating the dynamic changes were also not potential to be caused by viability of samples.

**FIGURE 3:**
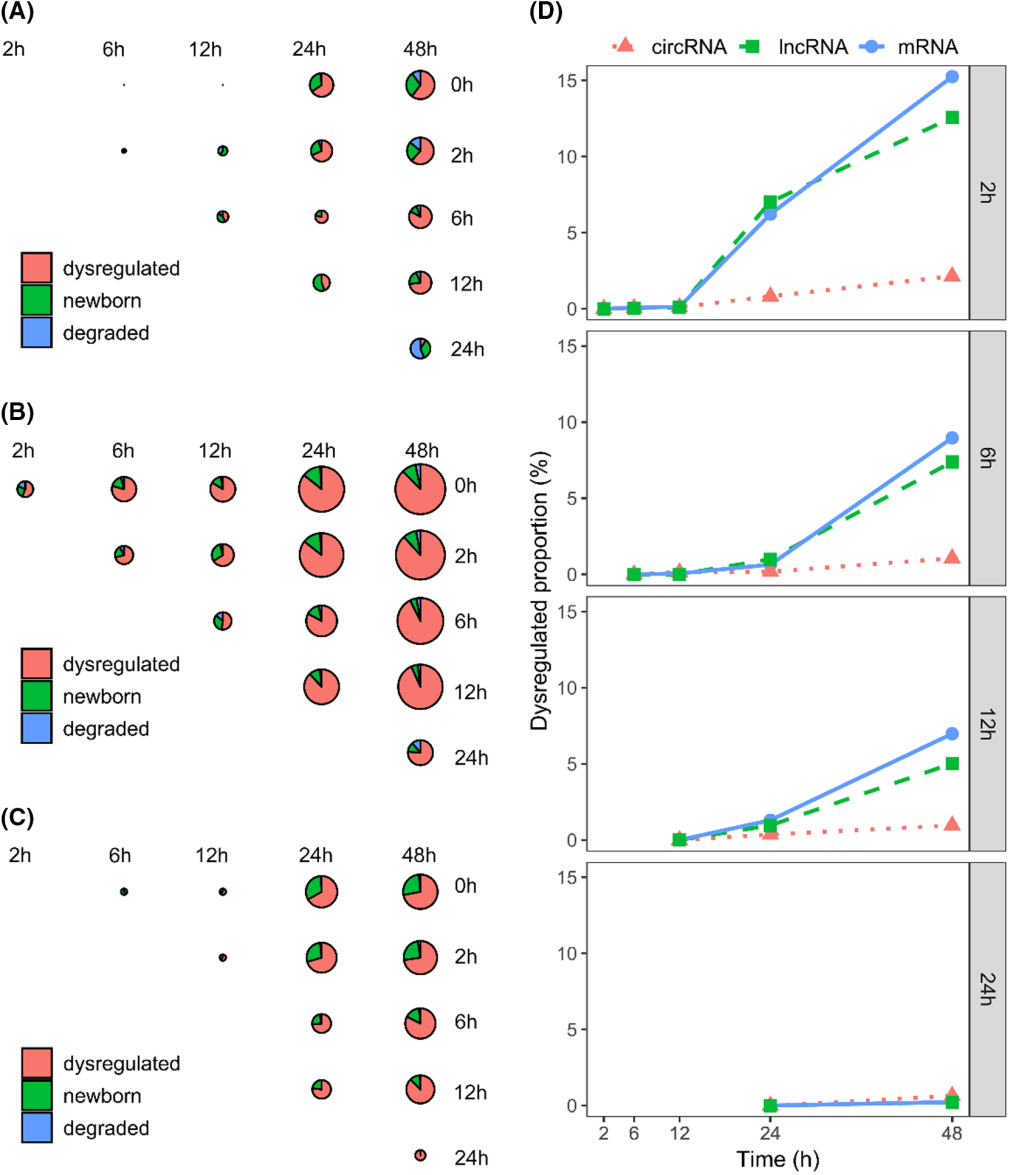
Dynamic gain or loss of circRNAs (A), mRNAs (B) and lncRNAs (C) upon incubation at different time points. Radius of each circle was the log_10_‐transformed number of dysregulated RNA transcripts between the time points at *x*‐axis and *y*‐axis. (D) The proportion of dysregulated transcripts at each time point against 2 h, 6 h, 12 h and 24 h, respectively, was also shown

Next, we looked into the dynamic gain or loss of both linear and circular RNA transcripts upon incubation at different time points. For each incubation time point (2 h, 6 h, 12 h, 24 h and 48 h), we identified the newborn or degraded transcripts, and calculated the number and proportion of these transcripts. We observed that the number of newborn or degraded transcripts increased gradually with the incubation time (Figure 3A–C). Comparing different types of RNA transcripts, we found that circular transcripts had smaller number of newborn and degraded transcripts than linear RNA transcripts (Figure 3A–C). This observation also exists when the dynamic gain or loss of RNA transcripts is normalized by the number of total transcripts in that class (Figure 3D). Furthermore, we found that the overlap of differential transcripts at certain time point with those in the previous time point was gradually increased as well (Figure S4). This indicates that the dynamic gain or loss of RNA transcripts is accumulated along the course of blood incubation.

### 3.3 Functional annotation of RNA dysregulation induced by blood handling delay

**FIGURE 4:**
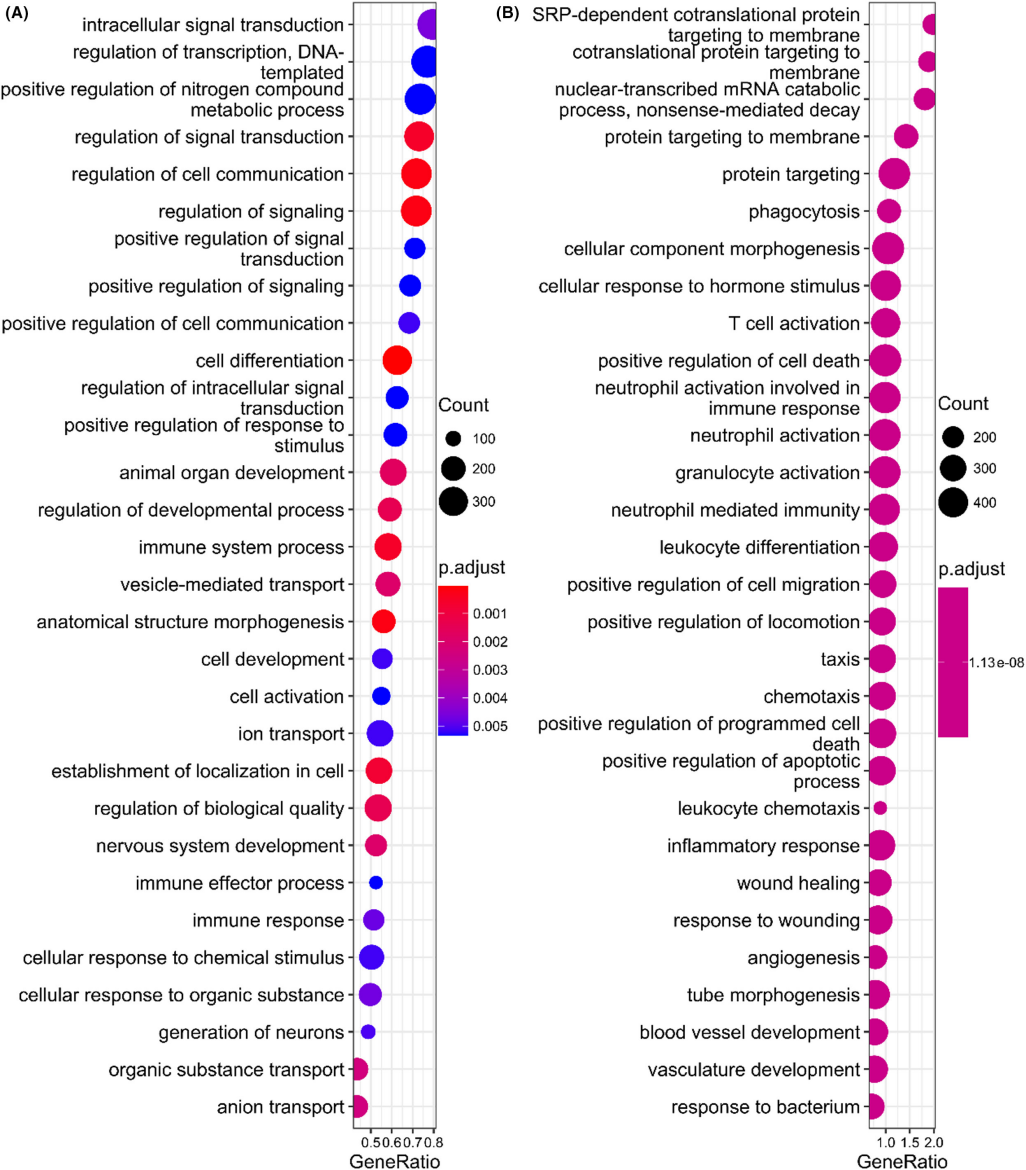
Functional annotation of dysregulated RNAs induced by blood handling delay at all time points: (A) The top 30 enriched biological pathways of the host genes of dysregulated circRNAs identified by *GSEA* (*p*‐value < .05), (B) the top 30 enriched biological pathways of dysregulated mRNAs identified by *GSEA* (*p*‐value < .05)

To deepen our understanding of the induced transcriptome changes resulting from prolonged incubation, we performed *GSEA* on dysregulated circRNA, mRNA and lncRNA genes at all five time points, respectively. While dysregulated lncRNA genes showed no significantly enriched BP, dysregulated circRNA and mRNA genes were enriched in several important BPs (Figure 4A,B). The enriched BPs of dysregulated circRNA genes upon incubation were mainly involved in three aspects (Figure 4A). First, several BPs that are related to signal transduction and communication were enriched, including ‘intracellular signal transduction’, ‘regulation of signal transduction’ and ‘regulation of cell communication’. Second, genes that are related to metabolic process are enriched, including ‘regulation of transcription, DNA templated’ and ‘positive regulation of nitrogen compound metabolic process’. Third, several development‐related BPs were included, such as ‘cell differentiation’ and ‘cell development’. Unlike circRNAs, incubation‐induced mRNA dysregulation showed distinct BPs, including co‐translational protein targeting related pathways, nonsense‐mediated decay (NMD) and immune response‐related pathways (Figure 4B). This suggests that circRNAs and mRNAs may perform different biological functions in response to external stimulus during sample handling delays. CircRNAs are likely to trigger signal cascade by acting as indirect regulators, while mRNAs tend to transport functional proteins to cell membrane and induce immune response by acting as direct executors. The co‐operation of circRNAs and mRNAs may mediate the structure morphogenesis, and even the apoptotic or death of blood cells.

### 3.4 Potential causes that induce circRNA dysregulation during incubation

**FIGURE 5:**
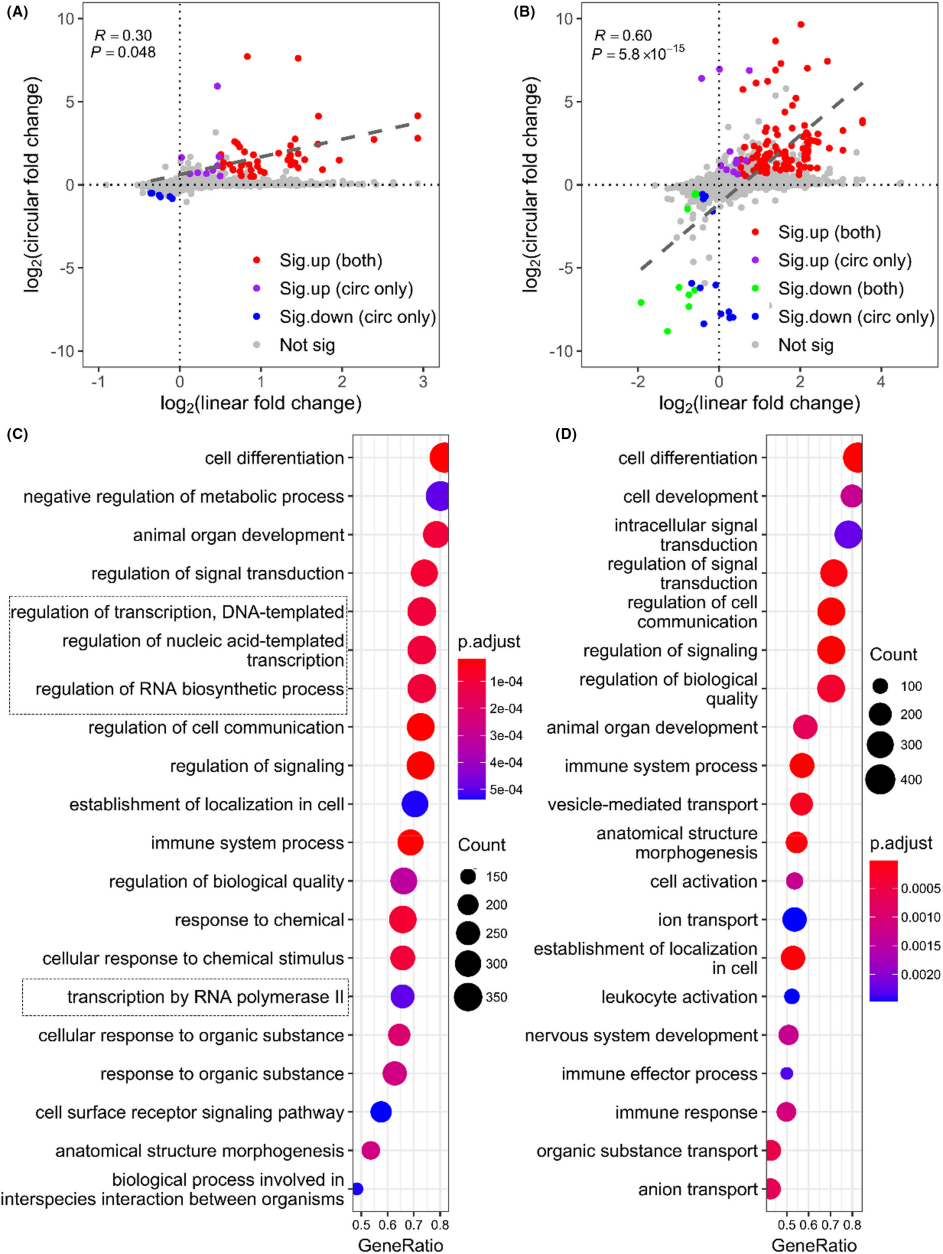
Correlation of log2(fold_change) of circRNAs versus log2(fold_change) of corresponding linear RNA expression at 24 h (A) and 48 h (B). Red and green dots represent transcription‐derived circRNAs that were upregulated or downregulated because of consistent upregulation or downregulation of their parental genes. Purple and blue dots represent splice‐derived circRNAs that were upregulated or downregulated whose parental genes showed no significant expression changes. Grey dots represent circRNAs that had no differential expression as well as their parental genes. The top 20 enriched biological pathways of the host genes of splice‐derived circRNAs (C) and transcription‐derived circRNAs (D) by *GSEA* (*p*‐value < .05) were also shown

CircRNA expression is the product of the transcribed expression level of circRNA host gene and its proportion of back‐splicing.^17^ Therefore, circRNA dysregulation during incubation can be caused by the dysregulated expression of its host gene and/or the dysregulated back‐splicing efficiency. To differentiate these two factors, we surveyed the correlation of expression changes of circRNAs and their corresponding genes at two time points (24 h and 48 h). We found a strong positive correlation between the expression changes of circRNAs and their corresponding host genes (Figure 5A,B). The majority dysregulated circRNAs, named transcription‐derived dysregulated circRNAs, were upregulated or downregulated owing to the upregulation or downregulation of their corresponding parental genes (Figure 5A,B, red and green dots). Notably, there were still some splice‐derived dysregulated circRNAs whose parental genes showed no significant expression changes (Figure 5A,B, purple and blue dots). The expression trends of representative circRNAs and their parental genes during incubation using TPM values, rather than fold change also confirmed the conclusions (Figure S5). *GSEA* analysis showed that biological functions of the host genes of splice‐derived dysregulated circRNAs (Figure 5C) had clear differences with those of transcription‐derived dysregulated circRNAs (Figure 5D). Interestingly, the splice‐derived dysregulated circRNA genes were enriched in several transcription‐related pathways, such as ‘regulation of nucleic acid‐templated transcription’ and ‘regulation of transcription by RNA *Pol II’*. This implied that the splice‐derived dysregulated circRNA genes were more likely to interact with RNA *Pol II* at promoter region and regulate gene transcription.

### 3.5 Alternative splicing of RNA transcripts during blood incubation

**FIGURE 6:**
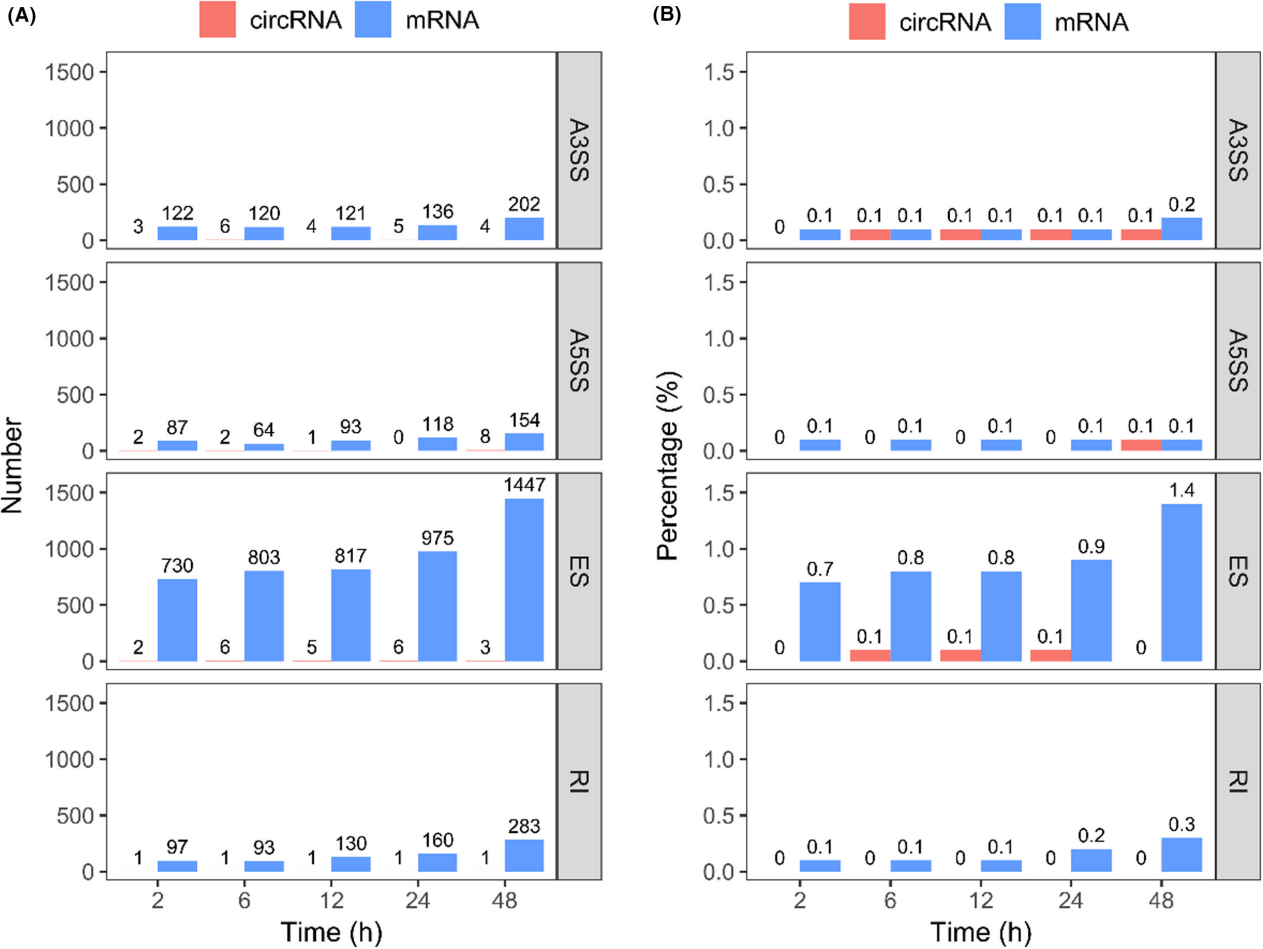
Sample handling delay induced differential AS events: (A) The number of differential AS events of circRNAs and mRNAs at different incubation time points; (B) The percentage of differential alternative splicing of circRNAs and mRNAs among the total identified AS circRNA or mRNA exons by *CIRI‐AS* or *rMATS‐turbo*

AS is one of the key processes of multi‐exonic gene expression during pre‐mRNA maturation, including skipped exon (ES), alternative 5′ splice site (A5SS), alternative 3′ splice site (A3SS) and retained intron (RI). Previous studies have found these events are common in circRNA formation as well.^28^, ^35^ To gain insights of incubation‐induced AS, we identified AS events of both circRNAs and mRNAs at each incubation time point and then performed differential alternative splicing analysis compared to the original time point (Figure 6). For linear mRNAs, we saw a gradual increase of all four AS events along the incubation course (Figure 6A). Comparing to linear mRNAs, circRNAs had a far smaller number of AS events upon blood incubation (Figure 6A). When normalized by the number of identified AS exons of each RNA type, the ratio of AS events occurred in circRNAs was still far lower than linear mRNAs (Figure 6B). This suggests that AS events of circRNA transcripts were more tolerated to handing delays than linear mRNAs. We further compared the distribution of parental genes that experienced differential AS events and observed significantly different size between circRNAs and mRNAs (Figure S6A), which might be explained by the longer circRNAs than mRNAs (Figure S6C). However, the expression abundance of circRNAs with differential AS events is statistically similar to that of mRNAs (Figure S6B), although circRNAs with AS events are more likely to have higher expression abundances than mRNAs (Figure S6D). These results suggested that the differences in detecting AS events were less likely to be relevant to the size and abundance of linear RNAs and circRNAs.

## 4 DISCUSSION

RNA expression in PBMCs or whole blood is important indicators of the host's immune status, and their aberrant expression is closely related to many disease conditions, creating favourable prospect for liquid biopsy.^36^ However, with accumulating evidences that blood transcriptome is sensitive to sample processing procedures, researchers have been concerning about the stability of blood RNAs and questioning robustness and reproducibility of RNA‐based biomarkers. Encouragingly, circRNAs open up another promising biomarker potential for human diseases due to their high stability, abundance and specificity.^16^, ^17^ However, knowledge remains elusive about the impact of prolonged incubation on the performance of circRNAs.

In this work, we first screened out dysregulated circRNAs, mRNAs and lncRNAs at six designed time points, and then compared the differences between circRNAs and linear mRNAs and lncRNAs. We observed gradual increase of expression value changes (Figure 2), transcript gain or loss (Figure 3) and differential alternative splicing (Figure 6) for linear mRNAs, lncRNAs and circular RNAs. Although some substantial changes have been observed for circRNA transcripts upon blood incubation, the variations of circRNA expressions were significantly smaller than those of linear mRNAs and lncRNAs. Our results suggested that the longest blood incubation time for linear mRNAs and lncRNAs at 4°C should be better controlled within 12 h. For circRNAs, the longest blood incubation time can be maintained at least 48 h. This not only convinced the higher stability and robustness of PBMC circRNAs over linear transcripts, but emphasized the necessity of excluding preanalytical artifacts before making conclusions regarding linear mRNAs and lncRNAs. Our conclusion is consistent to the results proposed in several previous studies that circRNAs are more stable^19^, ^32^ and have longer half‐lives^18^ than linear mRNAs. In this study, we focused on the effect of sample processing delay on circRNAs expression. To minimize the sample‐inherent biases, such as disease duration or severity, we used peripheral blood samples from healthy donors rather than patients. Although our study contains a relatively smaller number of 18 samples from 3 participants, our emphasis is on the variation between groups rather than inter‐individual variation. Therefore, it is appropriate to dissect expression changes of transcripts over time using three biological replicates at each time point.^37^ In contrast, Rochow et al. found gradually decreased circRNA expression values in clinical samples with RIN value reduction.^20^ This difference may be explained by the reduced RNA quality of clinical samples in their study, which may not be the case for the blood samples upon incubation (Figures 1A and S1). In another study, Savage et al. suggested that single cells were more active during sample incubation, and transcriptome alterations appeared earlier at the single‐cell level.^10^ Therefore, it is interesting to further analyze the dynamic changes of circRNA expression and evaluate its robustness at the single‐cell level.

In addition, we found that circRNA dysregulation was mainly derived from the dysregulated expression of their parental genes (Figure 5). However, *GSEA* analysis indicated that handling delay induced different changes between circRNA and mRNA transcripts (Figure 4), underscoring that circRNAs were not simple by‐products of their linear counterparts. Specifically, splice‐derived dysregulated circRNAs tended to perform their biological functions by interacting with *Pol II* (Figure 5C). Interestingly, some circRNAs have been experimentally validated to act as *Pol II* interactors. For example, *circEIF3J* and *circBPTF* can interact with U1 snRNP to form an RNA‐protein complex, and then bind to *Pol II* at the promoter region to enhance the transcription of their parental genes.^38^ *Ci‐ankrd52* can also associate with *Pol II* to regulate the expression of its parental gene by modulating the elongation of *Pol II*.^39^ These suggest blood incubation can cause distinct circRNA changes, although these changes may be neglectable even for samples with 48 h incubation.

Finally, AS events are the post‐transcriptional process to diversify transcriptome and proteome by adjusting incorporated exons or introns, which are ubiquitous in the formation of mRNAs and circRNAs.^29^, ^35^ Particularly, dysregulation of AS has been highly associated with human diseases, and is potential diagnostic biomarkers or therapeutics targets.^40^ Moreover, Dving et al. have proposed that sample incubation would induce isoform switch.^8^ Herein, we systematically investigated the effects of sample delays on four types of AS events for both circular RNAs and linear mRNAs. We found blood incubation resulted in profound impacts on AS of mRNAs, but not circRNAs (Figure 6). Therefore, it is imperative to take this technical bias into consideration when interpreting the results of differential AS analysis of blood mRNA transcripts. Meanwhile, we observed an enrichment of NMD‐related genes in differentially expressed mRNAs upon blood incubation (Figure 4B). Couple with the extensive mRNA AS events, NMD could be a post‐transcriptional mechanism in regulating gene expression upon incubation.^35^, ^41^ Specifically, we proposed that dysregulated AS may create isoforms with premature termination codons and truncated proteins under environmental stress, and indirectly participate NMD‐related pathways to accelerate cell death.

In summary, PBMC circRNAs have smaller transcriptome changes than mRNAs and lncRNAs upon sample processing delays, no matter the expression level or AS events. Therefore, circRNAs are superior to linear transcripts as the blood biomarker candidates, especially when the sample handling process of clinical blood samples cannot be normalized.

## AUTHOR CONTRIBUTIONS

**Guoxia Wen:** Data curation (equal); formal analysis (equal); methodology (equal). **Wanjun Gu:** Conceptualization (equal); data curation (equal); formal analysis (equal); funding acquisition (equal); investigation (equal); project administration (equal).

## CONFLICT OF INTEREST

The authors have no conflicts of interest to declare.

## Supporting information


Table S1

## Data Availability

The raw data of this study are available from the corresponding author upon reasonable request.
