# Supplementary material for: Circular RNAs in peripheral blood mononuclear cells are more stable than linear RNAs upon sample processing delay
Source: J Cell Mol Med. 2022 Aug 30;26(19):5021–32. doi: 10.1111/jcmm.17525 (PMC9549506; doi:10.1111/jcmm.17525)
Supplement: Supplementary file 1 — Table S1 [file JCMM-26-5021-s001.docx]

**Supplementary Table S1.** The number and percentage of differentially expressed circRNAs, mRNAs and lncRNAs at time points 2 h, 6 h, 12 h, 24 h and 48 h against time point 0 h.

|  | Time  (h) | Up-  regulated | Down-regulated | Up-regulated  (%) | Down-  regulated (%) |
| --- | --- | --- | --- | --- | --- |
| circRNAs | 2 | 0 | 0 | 0 | 0 |
|  | 6 | 0 | 1 | 0 | 0 |
|  | 12 | 0 | 1 | 0 | 0 |
|  | 24 | 55 | 6 | 1.1 | 0.1 |
|  | 48 | 117 | 24 | 2.3 | 0.5 |
| mRNAs | 2 | 9 | 7 | 0 | 0 |
|  | 6 | 63 | 9 | 0.2 | 0 |
|  | 12 | 72 | 8 | 0.2 | 0 |
|  | 24 | 2,384 | 292 | 7 | 0.9 |
|  | 48 | 4,080 | 2,178 | 11.9 | 6.4 |
| lncRNAs | 2 | 0 | 0 | 0 | 0 |
|  | 6 | 3 | 0 | 0.1 | 0 |
|  | 12 | 3 | 0 | 0.1 | 0 |
|  | 24 | 224 | 17 | 8.3 | 0.6 |
|  | 48 | 360 | 54 | 13.3 | 2 |


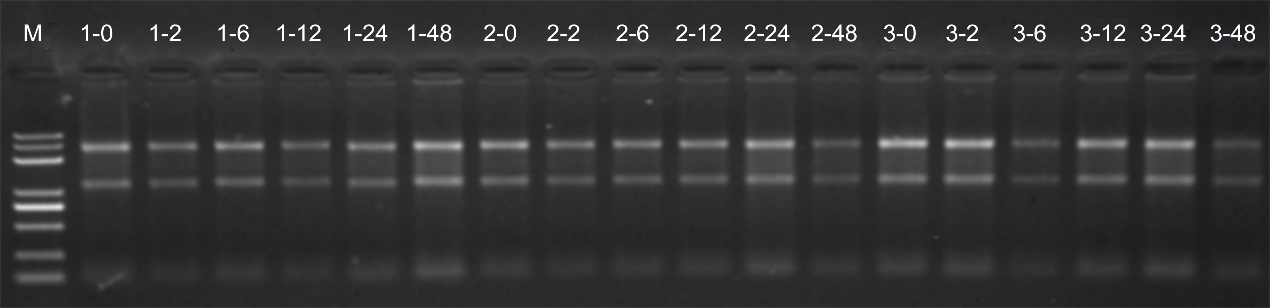


**Supplementary Figure S1.** RNA quality verification by 1% formaldehyde denaturing gel electrophoresis. M represents Marker Trans 2K Plus; Lane1-18 correspond to RNA solution of 0 h, 2 h, 6 h, 12 h, 24 h, 48 h for sample1, 0 h, 2 h, 6 h, 12 h, 24 h, 48 h for sample2, and 0 h, 2 h, 6 h, 12 h, 24 h, 48 h for sample3, respectively.


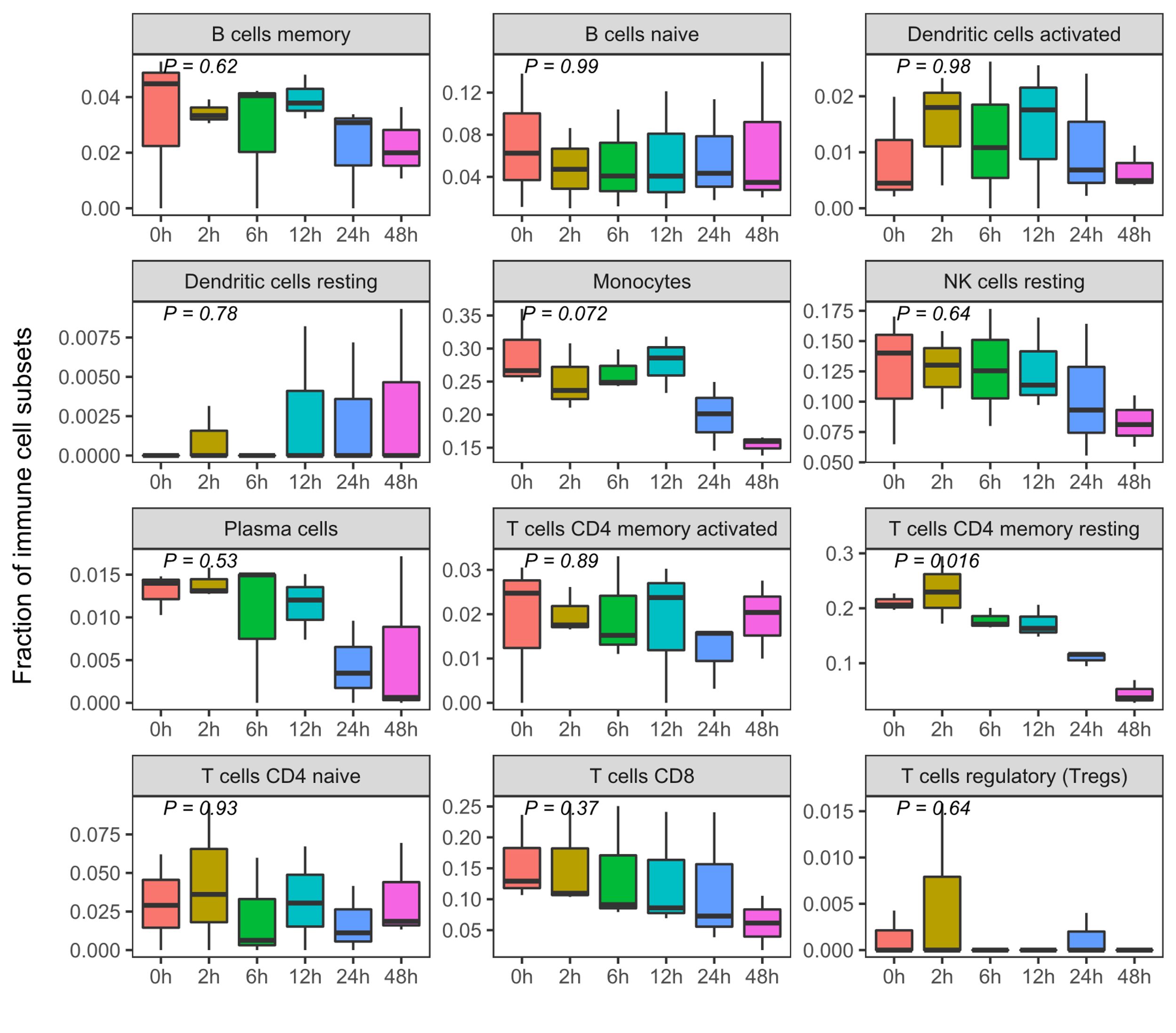


**Supplementary Figure S2.** The fraction of 12 immune cell subsets in PBMC samples at six time points. The *P* value was computed by *Kruskal-Wallis* test.


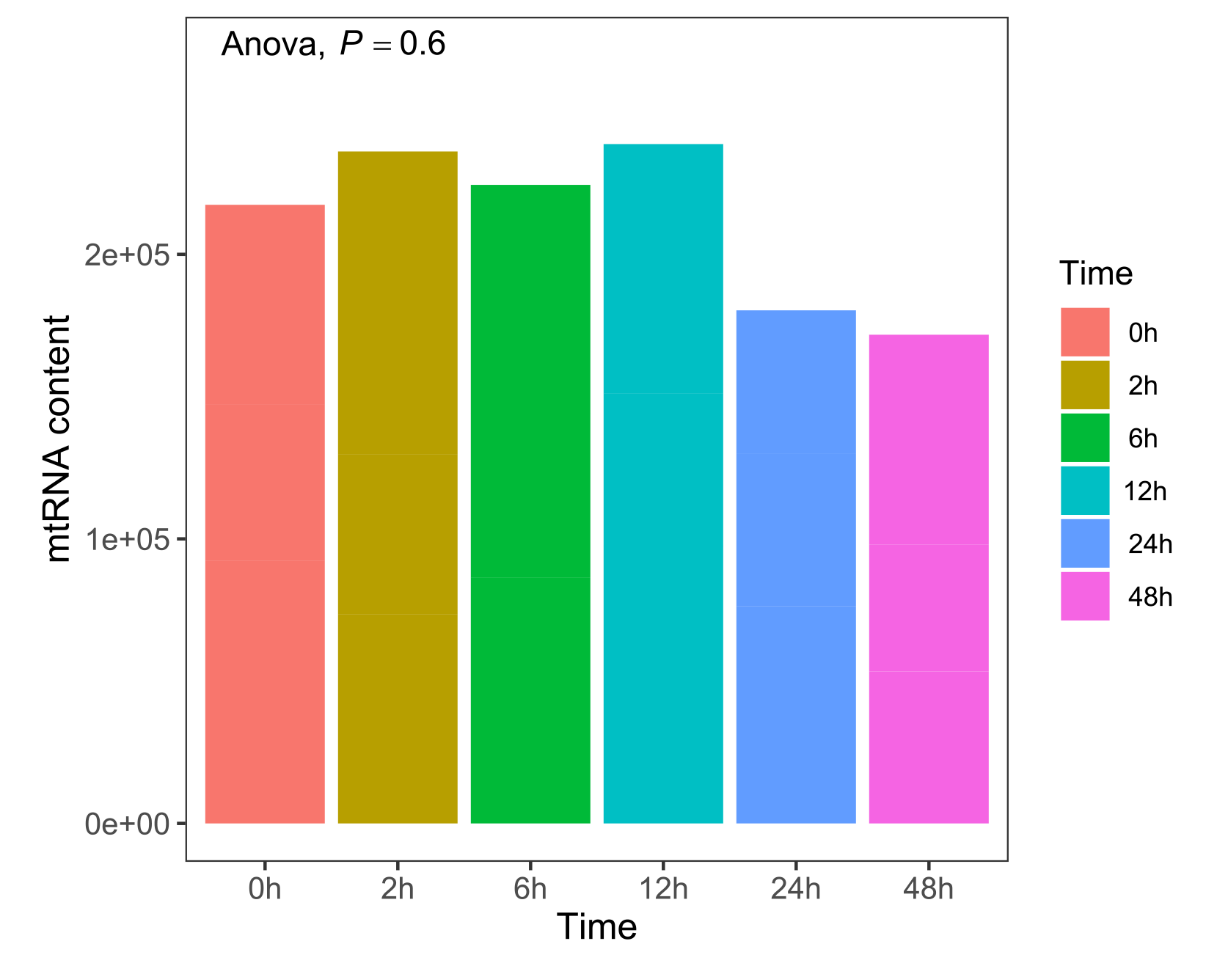


**Supplementary Figure S3.** The total content of mitochondrial RNAs of three samples at six time points. The *P* value was computed by *Anova* test.


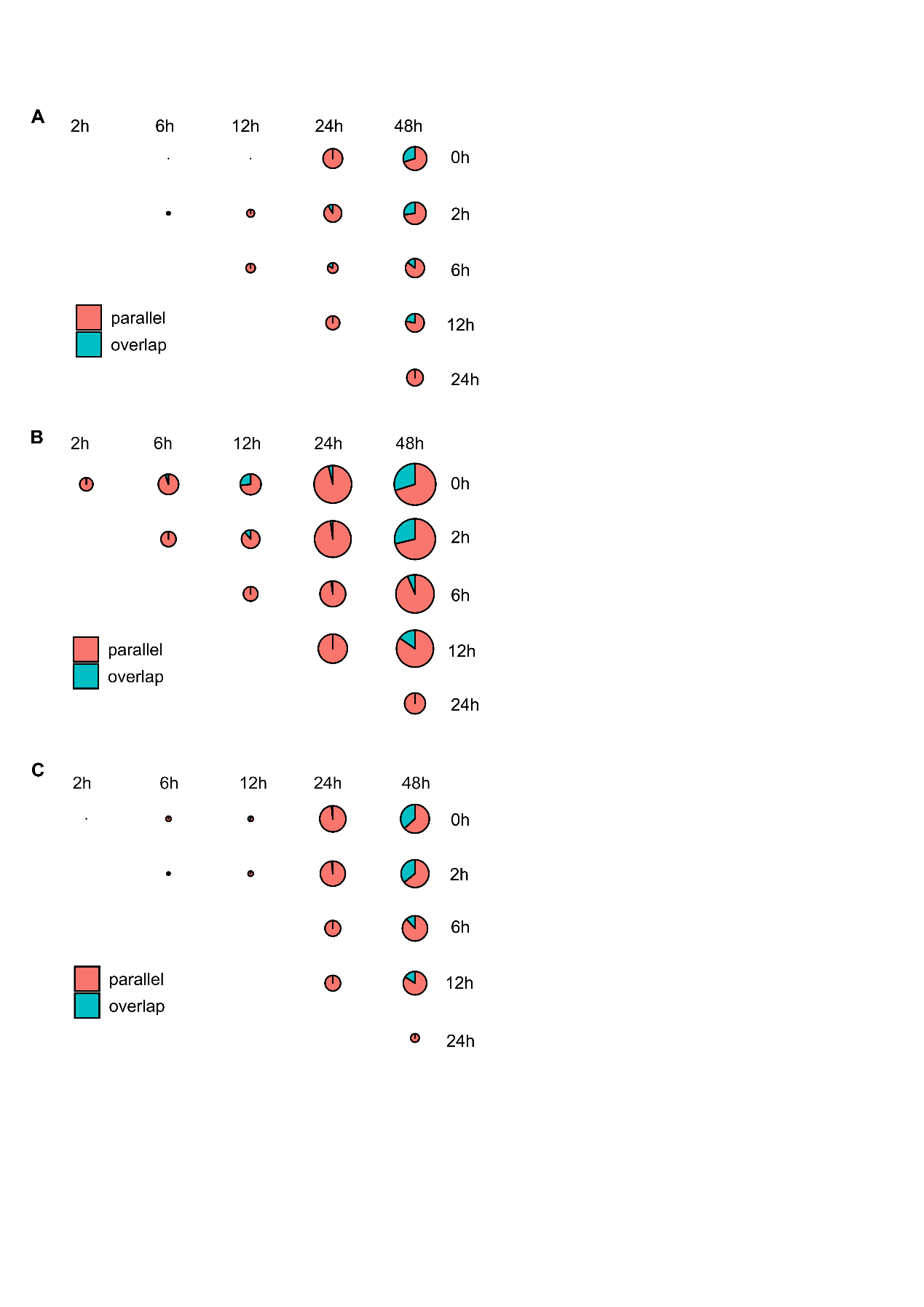


**Supplementary Figure S4.** The overlap of differentially expressed circRNAs **(A)**, mRNAs **(B)** and lncRNAs **(C)** at certain time points with those in the previous time point. Radius of each circle was the log_10_-transformed number of dysregulated transcripts between the time point at *x*-axis and the time point at *y*-axis.

**
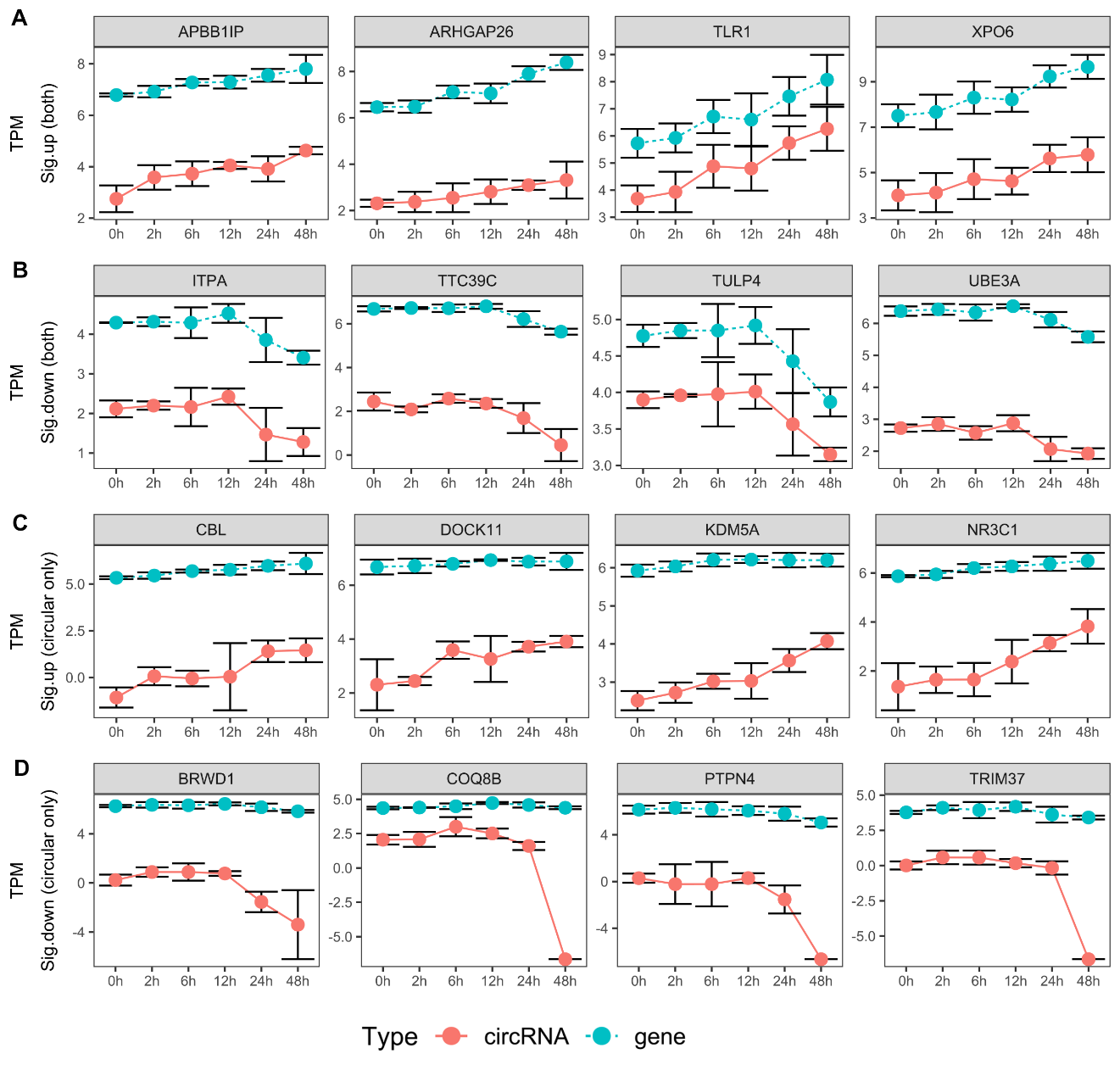
**

**Supplementary Figure S5.** Expression values of selected circRNAs and their parental genes at six time points in groups of sig.up (both) (A), sig.up (only) (B), sig.down (both) (C) and sig.down (only) (D).

**
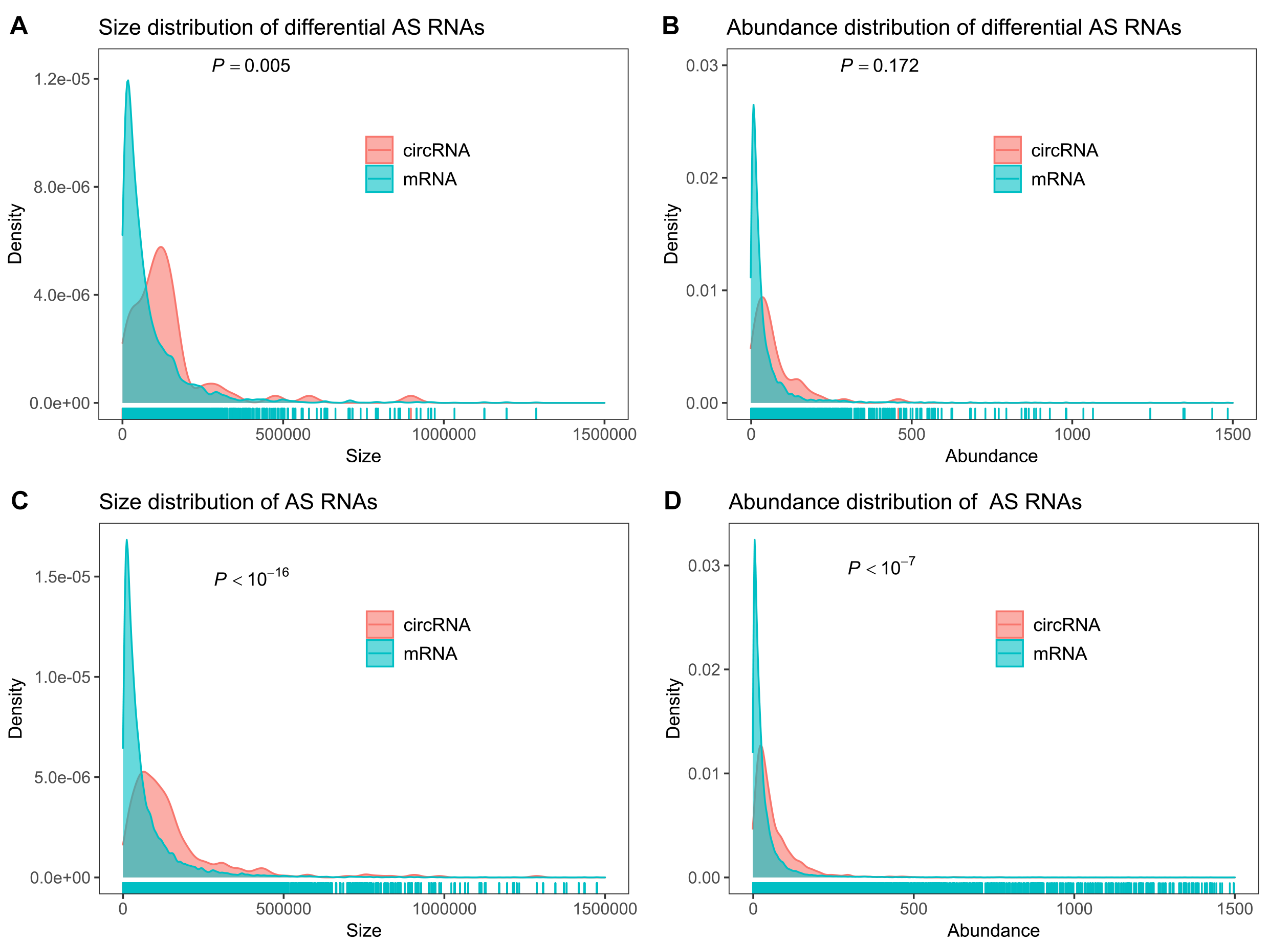
**

**Supplementary Figure S6.** Size distribution (A) and abundance distribution (B) of parental genes of differential alternative splicing circRNAs and mRNAs. Size distribution (C) and abundance distribution (D) of parental genes of all identified alternative splicing circRNAs and mRNAs. The *P* value was computed by *t* test.
